# Incidental Finding of Extreme Elevation of Serum Alkaline Phosphatase in Pregnancy

**DOI:** 10.7759/cureus.17211

**Published:** 2021-08-16

**Authors:** Beatriz Ferro, Inês Marques, Joana Paixão, Maria do Céu Almeida

**Affiliations:** 1 Obstetrics, Coimbra Hospital and University Center, Dr. Bissaya Barreto Maternity Hospital, Coimbra, PRT; 2 Faculty of Medicine, University of Coimbra, Coimbra, PRT; 3 University Clinic of Gynecology, Clinical Academic Centre of Coimbra, Coimbra, PRT; 4 Internal Medicine, Coimbra Hospital and University Center, Coimbra, PRT

**Keywords:** pregnancy complications, pregnancy, placenta diseases, chronic villitis, alkaline phosphatase

## Abstract

Increased levels of alkaline phosphatase (ALP) should alert us to changes in the liver, kidney, bone and malignancy. However, there is a physiological increase in pregnancy up to twice the upper limit. There has been a paucity of cases reporting extreme elevations of ALP in pregnancy. This is a case of an incidental pregnancy finding of a 24-fold increase in ALP in the third trimester (2877 U/L). The patient was kept under surveillance and ALP levels were monitored during the postpartum period.

Literature suggests a correlation between ALP elevation and several perinatal complications, proposing it could represent an important tool in monitoring high-risk pregnancies and underlying placental damage. We report a case with no perinatal complications and normal labor at term, with a placenta showing lesions of chronic villitis. We should not rely exclusively on an isolated, marked rise in ALP to dictate the approach in the absence of other fetomaternal considerations.

## Introduction

Alkaline phosphatase (ALP) is an enzyme produced by the liver, bones, small intestine, kidneys, and placenta, divided into isoforms and released into the bloodstream. During pregnancy, at the end of the second trimester, most of ALP activity comprises placental ALP isoenzymes (90% of which are P1 type and 10% P2 type) produced by syncytiotrophoblasts and appearing in maternal blood between the 15th and 26th weeks of pregnancy [1]. The role of placental ALP isoenzymes is not yet fully understood, but it is hypothesized that their major function is to aid in metabolism and facilitate transport across cell membranes.

Placental ALP isoenzymes plasma concentrations increase exponentially during gestation, usually not more than twice the pre-pregnancy upper limit of normal, returning to baseline in postpartum [2]. As extremely high ALP concentrations could suggest bone, hepatic, endocrine, renal diseases, and malignancies, they must be promptly excluded. Ideally, an elevated ALP can be fractionated to determine its origin.

Although there are currently few reports describing abnormally high serum ALP in pregnancy, some studies have shown that this enzyme could be an obstetric and perinatal marker associated with preterm delivery [3], hypertensive disorders [4], gestational diabetes [5], large for gestational age fetuses [6], and intrauterine growth restriction [7]. The exact mechanism of this association remains unknown, but it is hypothesized it may be the result of placental insufficiency [3]. It was described as an association between elevated ALP levels and placental damage from uteroplacental vascular disease, such as infarctions [8,9] or chronic intervillositis [10]. Nonetheless, there were studies that did not show any adverse perinatal outcomes with increased ALP levels [2,11].

We report a case of a pregnant woman with an incidental discovery of an extremely high isolated ALP serum concentration which had no fetomaternal outcomes, as levels returned to baseline in the postpartum.

This article was previously presented as a poster at the 27th European Congress of Perinatal Medicine on July 15, 2021.

## Case presentation

A 32-year-old female, with no relevant past medical history, presented in her second pregnancy. In the first pregnancy, she had a 36 weeks preterm labor after a preterm rupture of membranes, without further complications.

At 37 weeks, elevated blood pressure prompted urgent request for analyzes that revealed an extremely isolated increase in ALP of 2877 U/L (normal range 30-120 U/L), with no previous values other than a 159 U/L in 2015 during the first pregnancy. There were no complaints nor a previous history of bone, liver, or renal disease.

Laboratory tests showed normal hepatic, endocrine, and renal functions, without proteinuria. Tumor markers (cancer antigen 125 {CA-125}, carcinoembryonic antigen, alpha-fetoprotein, beta-human chorionic gonadotropin {beta-hCG}, and cancer antigen 19-9 {CA-19-9}) were non-pathological. She was discharged with recommendations of blood pressure monitoring and was scheduled for routine prenatal follow-up care in the following week. ALP isoenzyme differentiation revealed a predominance of placental isoenzyme (97%), with both liver and bone isoenzymes within the normal range.

**Figure 1:**
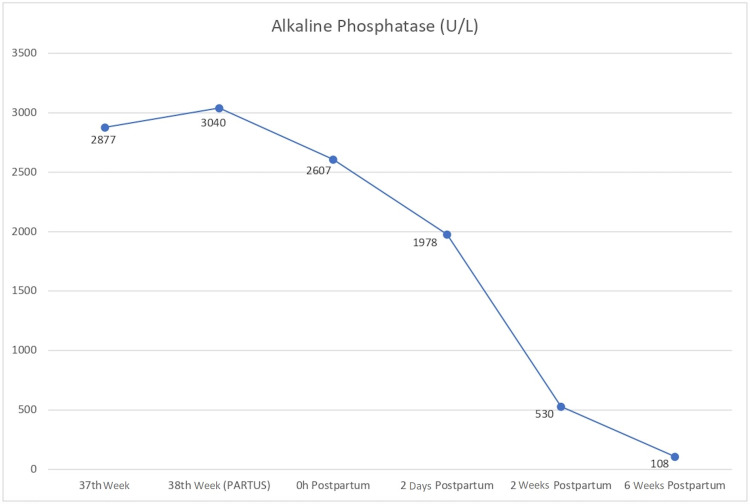
Changes in total serum alkaline phosphatase concentrations during pregnancy and postpartum period - reached a peak at the 38th gestational week. After birth, it decreased exponentially during the following weeks, and became normal six weeks later.

On her next appointment, one week later, she maintained grade I hypertension and presented in labor. ALP raised to 3040 U/L (25-fold increase in upper normal limit). She had an uneventful vaginal delivery and a mature normal-weight baby boy was born, with 3360 g, and a 9/10/10 appearance, pulse, grimace, activity, and respiration (APGAR) score. They were discharged two days following delivery. Placental ALP levels were measured immediately after delivery (2607 U/L), at two days (1978 U/L), two weeks (530 U/L) postpartum, and returning to normal at the sixth week postpartum (108 U/L) (Figure 1).

**Figure 2:**
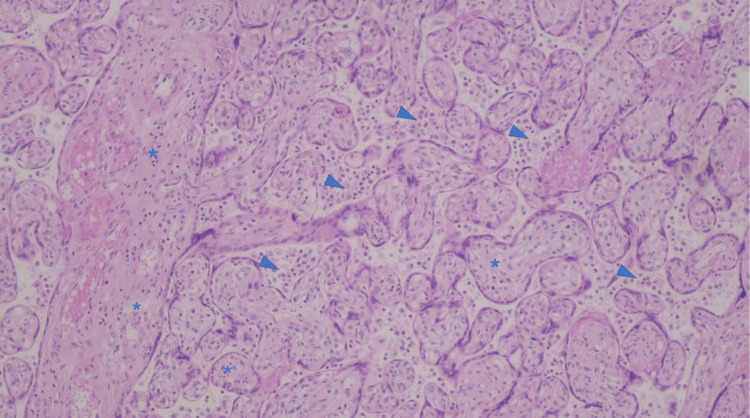
Anatomopathological evaluation of the placenta: placenta of the third gestational trimester with lesions of intervillositis (arrowhead) and chronic minimal villi, multifocal (asterisk).

The placenta showed a 25th percentile weight revealing a suitable 38 weeks development and minimal chronic villitis (Figure 2).

The male infant is doing well at five months of age and has reached all developmental milestones.

## Discussion

If a patient has an isolated elevation of ALP, the first step in the evaluation, if possible, is to confirm its origin since it can come from various sources. If there are abnormalities in other liver chemistries, confirmation is typically not required. Based on its connection to numerous malignant diseases, tumor markers evaluation is mandatory [12].

As stated earlier, previous studies tried to understand the mechanism behind this abnormal elevation, which is not fully understood. It was suggested a correlation between ALP elevation and several perinatal complications such as preterm delivery [3], gestational diabetes [5], large for gestational age fetuses [6], and intrauterine growth restriction [7].

In a retrospective study by Wilkof-Segev et al., it was made a review of cases with an extreme elevation of ALP in pregnancy during the period of eight years. They concluded that the obstetric complications were higher, with 57% of the cases registering some perinatal complication during pregnancy, namely hypertension, gestational diabetes, and preterm delivery. Most of the studied placentas revealed some pathological alteration, such as vasculopathy or inflammation [13]. Similarly, other studies have shown that chronic histiocytic intervillositis, a rare placental pathology, is associated with high obstetric morbidity, namely spontaneous abortion and intrauterine growth restriction, and ALP has been identified as an early marker for these lesions. Its management is still controversial, and recurrence in future pregnancies is a possibility [10].

In our case, anatomopathological findings described signals of intervillositis and chronic villi, although minimal, that have been previously associated with raised ALP levels, suggesting it could be used as an early marker of placental lesions [10,14].

It has been proposed that routine measurement of ALP levels could be an important tool in monitoring high-risk pregnancies and underlying trophoblastic damage, thus preventing the worst outcomes [7,15]. However, other studies have shown that this parameter isolated increase does not imply maternal-fetal complications, as was observed in the present case [11]. Also, there are no reported cases of fatal complications.

## Conclusions

In this patient, there was an incidental discovery of an extreme elevation of ALP, without further repercussions for both mother and baby during pregnancy and after delivery.

In the presence of an extreme ALP elevation during pregnancy, there should be a systematic approach to exclude its different etiologies. Regular maternal-fetal surveillance, subsequent placenta anatomopathological analysis, and ALP levels monitoring must be performed until its normalization.

Furthermore, we should not rely exclusively on an isolated, marked rise in serum ALP to dictate the approach if other fetomaternal considerations are absent.

## References

[1] Nozawa S, Arai H, Jeng C (1984). Shift of placental alkaline phosphatase isoenzymes in the course of pregnancy. [Article in Japanese]. Nihon Sanka Fujinka Gakkai Zasshi.

[2] Boronkai A, Than NG, Magenheim R (2005). Extremely high maternal alkaline phosphatase serum concentration with syncytiotrophoblastic origin. J Clin Pathol.

[3] Ferianec V, Linhartová L (2011). Extreme elevation of placental alkaline phosphatase as a marker of preterm delivery, placental insufficiency and low birth weight. Neuro Endocrinol Lett.

[4] Rajagambeeram R, Raghavan SA, Ghosh S, Basu S, Ramasamy R, Murugaiyan SB (2014). Diagnostic utility of heat stable alkaline phosphatase in hypertensive disorders of pregnancy. J Clin Diagn Res.

[5] Lozo S, Atabeygi A, Healey M (2016). Extreme elevation of alkaline phosphatase in a pregnancy complicated by gestational diabetes and infant with neonatal alloimmune thrombocytopenia. Case Rep Obstet Gynecol.

[6] Liu Y, Hou W, Meng X (2018). Early elevated alkaline phosphatase increases the risk of large-for-gestational-age birth weight in pregnant women with normal glucose tolerance. Diabetes Res Clin Pract.

[7] McErlean S, King C (2019). Does an abnormally elevated maternal alkaline phosphatase pose problems for the fetus?. BMJ Case Rep.

[8] Collins PA (1981). Serum constituents in pregnancy including 4 cases with elevated alkaline phosphatase levels. Clin Biochem.

[9] Davis CJ, Booth J, Summerfield J, Lazda EJ, Regan L (1999). Grossly elevated placental derived alkaline phosphatase in pregnancy as a marker for uteroplacental vascular disease. J Obstet Gynaecol.

[10] Ferreira M, Fonseca AG, Cunha V, Almeida MM, Santos A, Ilgenfritz R (2017). Chronic histiocytic intervillositis: a rare cause of placenta mediated obstetrical events associated with isolated alkaline phosphatase elevation? [Article in Portugese]. Med Interna.

[11] Stanley Z, Vignes K, Marcum M (2020). Extreme elevations of alkaline phosphatase in pregnancy: a case report. Case Rep Womens Health.

[12] Nozawa S, Udagawa Y, Ohkura H (1990). Serum placental alkaline phosphatase (PLAP) in gynecologic malignancies—with special reference to the combination of PLAP and CA54/61 assay. Clin Chim Acta.

[13] Wilkof-Segev R, Hallak M, Gabbay-Benziv R (2021). Extremely high levels of alkaline phosphatase and pregnancy outcome: case series and review of the literature. J Perinat Med.

[14] Parant O, Capdet J, Kessler S, Aziza J, Berrebi A (2009). Chronic intervillositis of unknown etiology (CIUE): relation between placental lesions and perinatal outcome. Eur J Obstet Gynecol Reprod Biol.

[15] Ranganath L, Taylor W, John L, Alfirevic Z (2008). Biochemical diagnosis of placental infarction/damage: acutely rising alkaline phosphatase. Ann Clin Biochem.

